# Supplementary material for: Highly accurate long reads are crucial for realizing the potential of biodiversity genomics
Source: BMC Genomics. 2023 Mar 16;24:117. doi: 10.1186/s12864-023-09193-9 (PMC10018877; doi:10.1186/s12864-023-09193-9)
Supplement: Supplementary file 1 — Additional file 1: Figure S1. Annotated h-fibroin genes for Hesperophylax magnus assembled with two long-read data sets and visualized in Geneious. Table S1. A complete list of the genome assemblies and associated metadata used in this study included as a separate Microsoft Excel file. [file 12864_2023_9193_MOESM1_ESM.zip › BMC Genomics (Supp. Materials).docx]

**SUPPLEMENTARY MATERIALS**

**Highly accurate long reads are crucial for realizing the potential of biodiversity genomics**

Scott Hotaling*, Edward R. Wilcox, Jacqueline Heckenhauer, Russell J. Stewart, and Paul B. Frandsen*

***Authors for Correspondence:**

Scott Hotaling, Department of Watershed Sciences, Utah State University, Logan, UT, USA; Email: [scott.hotaling1@gmail.com](mailto:scott.hotaling1@gmail.com); Phone: (828) 507-9950

Paul B. Frandsen, Department of Plant and Wildlife Sciences, Brigham Young University, Provo, UT, USA; Email: [paul_frandsen@byu.edu](mailto:paul_frandsen@byu.edu); Phone: (804) 422-2283

**Figures:**


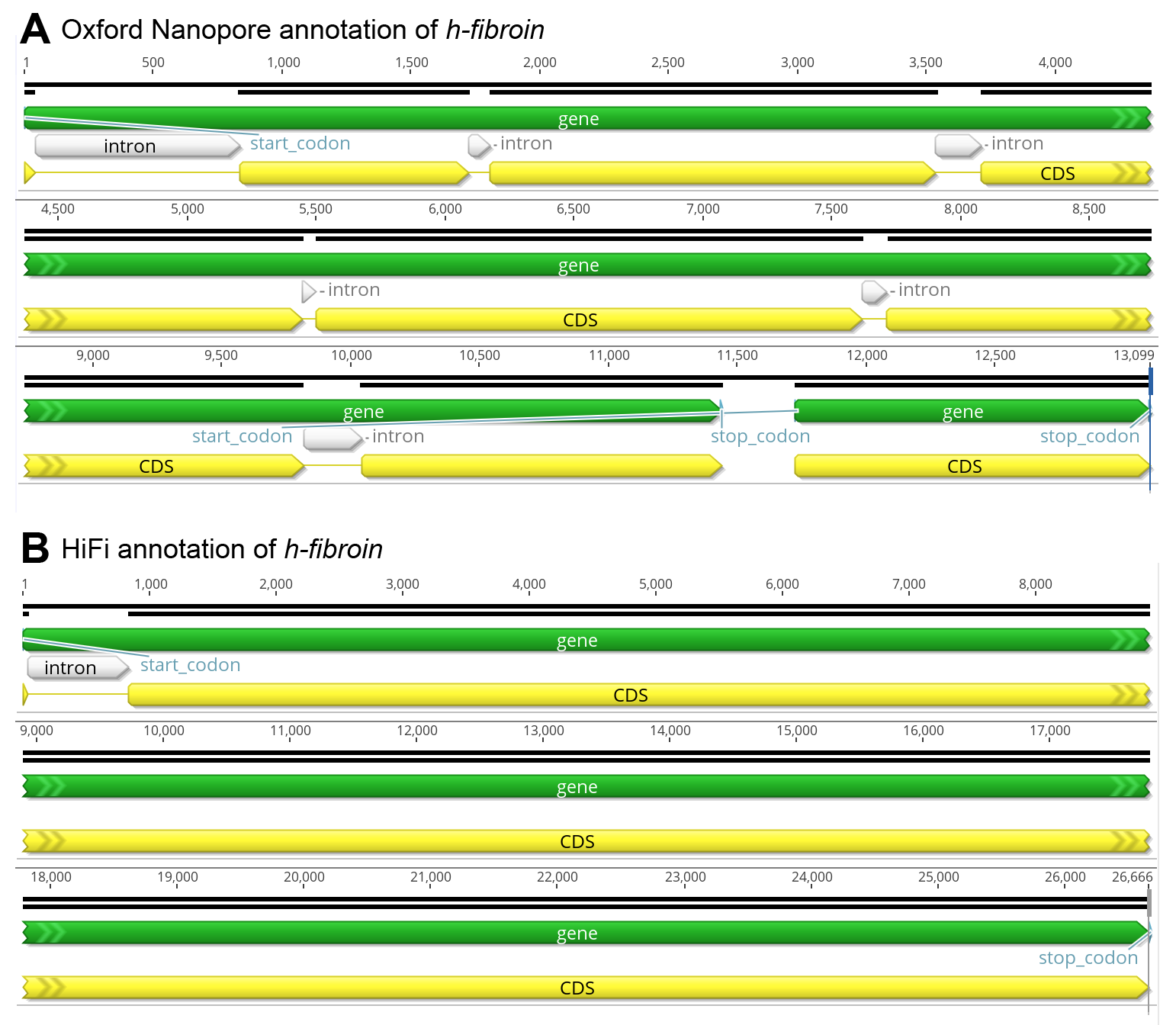


**Figure S1.** Annotated *h-fibroin* genes for *Hesperophylax magnus* assembled with two long-read data sets and visualized in IGV.

**Tables:**

**Table S1.** A complete list of the genome assemblies and associated metadata used in this study included as a separate Microsoft Excel file.
